# Comparison of Safety Profile of Semaglutide, Tirzepatide, and Liraglutide in Obese Patients: FAERS Database Investigation

**DOI:** 10.51894/001c.143624

**Published:** 2025-10-01

**Authors:** Yash P Ashara, Esha Garg, Marc Feldman

**Affiliations:** 1 Internal Medicine Detroit Medical Center Sinai Grace Hospital; Wayne State University

## INTRODUCTION

Obesity is a significant global health concern, linked to various chronic diseases and elevated healthcare costs. GLP-1 receptor agonists like Semaglutide, Tirzepatide, and Liraglutide are prominent for their efficacy in weight reduction and metabolic improvement. They work by enhancing glucose-dependent insulin secretion and suppressing appetite.

## OBJECTIVE

Our study seeks to assess the safety profiles of the GLP-1 receptor agonists Semaglutide and Tirzepatide against the established agent Liraglutide. We aim to better understand their relative safety in terms of serious events, hospitalizations, deaths, and various adverse reactions, including gastrointestinal, psychiatric, and nervous system disorders.

## METHODS

The US Food and Drug Administration’s Adverse Event Reports System (FAERS) database was queried for Semaglutide, Tirzepatide, and Liraglutide in obesity. Demographics of adult patients were recorded along with adverse reactions on these drugs. Analysis was performed using risk ratio (RR) with 95% confidence interval (CI) and statistical significance P < 0.05.

## RESULTS

A dataset of 1713 reported adverse event cases of obese patients treated with Semaglutide, Tirzepatide, and Liraglutide until December 31, 2024, was extracted from the FAERS database. 75.9% were female. Patients on Tirzepatide experienced a significantly higher risk of hospitalizations (RR 3.13, 95% CI: 2.75-3.55) and serious events (RR 1.15, 95% CI: 1.04-1.29) compared to Liraglutide users. Semaglutide also exhibited increased serious events (RR 1.21, 95% CI: 1.12-1.31). Gastrointestinal disorders (RR 0.82) and general disorders (0.8) were significantly low in Semaglutide when compared to Liraglutide. Tirzepatide had significantly lower risk of hepatobiliary (RR 0.14), infections (RR 0.45) and gastrointestinal (RR 0.5) disorders when compared to Liraglutide. The occurrence of eye, skin, psychiatry, nervous disorders and injury were significantly higher in Semaglutide, while there was no statistical difference in eye and nervous disorders in Tirzepatide. There was no statistical significance between rates of hepatobiliary and infections in Semaglutide and Liraglutide. Incorporating gender did not yield any statistical significance.

## DISCUSSION

The elevated risks of hospitalizations associated with Tirzepatide suggest a concerning safety profile that merits cautious use and continuous monitoring. Although Semaglutide and Tirzepatide provide substantial metabolic benefits, their adverse effect profiles require careful patient selection and post-marketing surveillance to optimize treatment strategies.

